# A novel remote assessment pathway to streamline the management of two-week-wait suspected head and neck cancer referrals: a prospective analysis of 660 patients

**DOI:** 10.1017/S002221512400015X

**Published:** 2024-06

**Authors:** Christopher Metcalfe, Soo Oh, Nina Glazzard, Elizabeth Ross, Ajith George

**Affiliations:** Department of Otolaryngology, Royal Stoke University Hospital, University Hospitals North Midlands, Keele University, Stoke-on-Trent, UK

**Keywords:** head and neck cancer, telemedicine, technology, coronavirus disease 2019

## Abstract

**Objective:**

This study analyses outcomes for 660 patients managed via a novel telescopic pathway for suspected head and neck cancer referrals.

**Method:**

Data were collected prospectively between January 2021 and December 2022, capturing all two-week-wait referrals triaged as low risk and managed via a nurse-led clinic for nasendoscopic examination and consultant-led remote assessment.

**Results:**

In total, 660 patients were included. There were six head and neck cancers diagnosed, giving a conversion rate of 0.9 per cent. Mean (standard deviation) time to informing the patient whether they did or did not have cancer (28-day faster diagnosis standard) was 28.6 days (20.2), with no significant difference observed in patients imaged prior to review (*p* = 0.63). No missed cancers were detected in the follow-up period.

**Conclusion:**

Low-risk head and neck cancer referrals can be safely managed in a nurse-led clinic for recorded examination with asynchronous consultant-led management. Further work is required to ensure adherence to the new faster diagnosis standard.

## Introduction

The novel coronavirus disease 2019 (Covid-19) pandemic has shaped the current state of healthcare in the UK. During the initial wave of the pandemic, the assessment and management of suspected head and neck cancer patients were profoundly impacted, with the British Association of Head and Neck Oncologists issuing a position statement on delivering head and neck cancer care during the pandemic.^1^ The focus at that time was on the identification and prioritisation of high-risk patients likely to have a malignancy, and minimising hospital exposure in patients who were frail or those with significant co-morbidities.

Post-pandemic, pressures on the head and neck cancer pathway remain high and this has been compounded by the introduction of a new Faster Diagnosis Standard, introduced in April 2020, to ensure that all patients who are referred for the investigation of suspected cancer find out within 28 days if they do or do not have a cancer diagnosis.^2^ These unprecedented pressures on the head and neck cancer pathway have served as a stimulus for the development of new methods of service delivery, including the implementation of telemedicine to triage, assess and manage patients.

A novel telescopic pathway has been implemented at our unit which utilises high-quality mobile imaging alongside secure store-and-forward technology to streamline the two-week-wait head and neck cancer pathway.^3^ This pathway is based on the risk stratification of patients using a widely validated risk calculator,^4^ whereby high-risk patients are still seen face-to-face by a consultant head and neck surgeon, in keeping with the traditional out-patient model of care. However, low-risk patients are diverted to a telescopic clinic led by a trained nurse practitioner, where a flexible nasendoscopy and oral examination are performed and recorded, with the videos reviewed remotely and asynchronously by a consultant head and neck surgeon before an outcome is communicated to the patient via text message.

A pilot study^3^ of this pathway suggested that it was safe for patients, with potential benefits such as consultant-led care for all patients, enhanced documentation and optimisation of consultant time, whilst also providing some flexibility within the pathway to accommodate fluctuating demand. This is the first pathway of its kind within the National Health Service (NHS), and we are now able to present a long-term analysis of all patients assessed and managed via this novel pathway.

## Materials and methods

The study was carried out in a UK secondary/tertiary referral unit and involved a prospective service analysis. It was prospectively registered as a service evaluation and approved by our institutional review board.

### Description of novel pathway

All two-week-wait referrals are triaged based on a written referral from a general practitioner. Unexplained neck lump referrals are diverted directly for imaging prior to review and all other referrals are stratified into high and low risk based on a validated head and neck cancer risk calculator (HaNC-RC v.2, http://www.orlhealth.com/risk-calculator-2.html).4

An estimated probability of head and neck cancer of more than 7.1 per cent is considered high risk. High-risk patients are seen face-to-face by a head and neck consultant as per a traditional out-patient model. This includes patients with potentially sinister findings on imaging, if diverted prior to review.

Low-risk patients are diverted into a telescopic clinic led by a trained nurse practitioner and reviewed as soon as possible. The majority of these patients are reviewed by the nurse practitioner in a hospital clinic, but some patients are reviewed in a community clinic. During this consultation, the patient undergoes a repeat face-to-face risk stratification based on the above calculator, and a flexible nasendoscopy and oral examination are performed and recorded using store-and-forward technology. The scoring and examination are then reviewed asynchronously and remotely by a consultant head and neck surgeon who then communicates an outcome to the patient. This arm of the pathway is also used for patients who require nasendoscopy after being diverted for imaging, where the imaging has not demonstrated any serious pathology.

### Equipment

**Figure 1.**
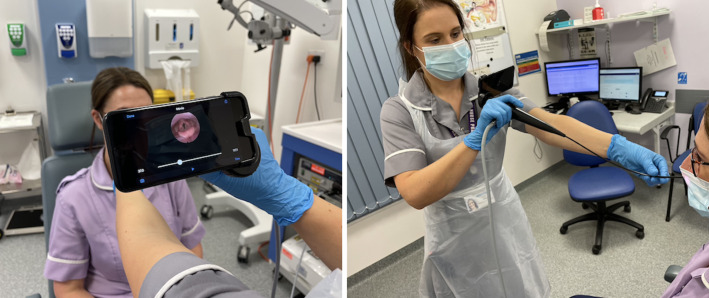
Equipment used on the telescopic pathway.

All images were acquired using a standard fibre-optic flexible nasendoscope attached to a secured iPhone SE2 (Apple Inc., California) using an endoscope–smartphone adapter (endoscope-i Ltd, UK) (Figure 1). Images were viewed, recorded and stored using the e-i Pro application (endoscope-i Ltd, ) in accordance with trust protocol. Videos stored on this device were accessed by the reviewing consultant using a corresponding iPad (8th generation; Apple Inc.) equipped with bespoke software that allows the user to choose and store stills from the video and generate a summary document including four stills alongside a diagnosis and outcome.

**Figure 2.**
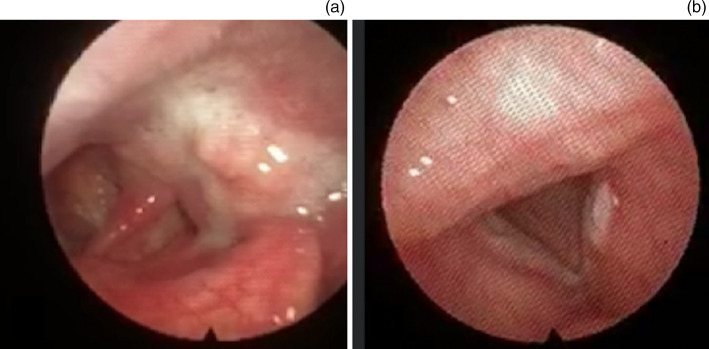
Examples of two cancers picked up in the low-risk clinic. (a) Shows a left piriform fossa squamous cell carcinoma; (b) shows a left vocal cord squamous cell carcinoma.

### Sampling

All patients placed on the low-risk telescopic referral pathway following triage between January 2021 and December 2022 were included. Most patients were new referrals from general practitioners, but there were also low-risk patients brought into the nurse-led clinic for flexible nasendoscopy having been diverted for imaging prior to review. All referral sources were included. Patients seen face-to-face on the high-risk arm of the pathway were excluded.

All patients on the pathway were recorded prospectively, with subsequent retrospective collection of follow-up data (e.g. re-referrals) using the digital patient record system at our unit. Data were collected on patient demographics, referral source, waiting times, time between telescopic clinic and consultant review of images, time to diagnosis and clinical outcomes. Time to diagnosis was recorded with reference to the 28-day Faster Diagnosis Standard, whereby the endpoint was either a diagnosis of cancer or the point at which the patient was informed that they were being removed from the cancer pathway.

### Data analysis

All data were entered into a standardised spreadsheet for analysis. A descriptive analysis was performed for patient demographics, referral numbers, waiting times and clinical outcomes. An independent *t*-test was used to compare the Faster Diagnosis Standard for patients diverted for a scan prior to review versus those reviewed directly, and for those seen in a hospital versus a community clinic. Statistical analysis was performed using IBM SPSS Statistics (version 29).

## Results

A total of 660 patients were seen on the low-risk pathway between January 2021 and December 2022. There were 541 females (82 per cent) and 119 males (18 per cent), with a mean age of 58 years (range, 19–96 years). The mean follow up for this group was 15 months (range, 3–27 months). In total, 543 patients (82.3 per cent) were triaged as low-risk and seen directly in a nurse-led clinic for nasendoscopy, while 117 patients (17.7 per cent) had a ‘neck lump’ mentioned in the referral and were therefore diverted for a scan before being brought back for nasendoscopy.

Overall, there were six head and neck cancer diagnoses, giving a cancer conversion rate of 0.91 per cent (Table 1). Of the patients booked directly into the nurse-led clinic for endoscopy, the mean waiting time from referral to review was 21 days (range, 0–79 days). The remote images captured were deemed adequate for assessment in 98.9 per cent of cases, with only seven patients (1.1 per cent) requiring a further examination due to a non-diagnostic initial telescopic examination. None of these seven patients were diagnosed with cancer.

**Table 1.** Patients with a head and neck cancer diagnosis, alongside initial triage outcome (based on written referral) and corresponding HaNC-RC prediction score (calculated by nurse practitioner at face-to-face review)

Age	Gender	Triage outcome from written referral	HaNC-RC v.2 score from face-to-face nurse review (%)	Diagnosis
**36**	Female	Low risk	2.53	SCC larynx
**30**	Female	Low risk	0.10	Vocal fold high-grade dysplasia
**73**	Female	Low risk	1.7	SCC piriform fossa
**47**	Female	Low risk	61.59*	SCC supraglottic
**78**	Female	Low risk	3.57	SCC larynx
**75**	Female	Low risk	0.70	SCC larynx

* Referred otalgia and throat pain not mentioned on written referral hence initial low-risk triage. SCC = squamous cell carcinoma

The mean time from remote examination to consultant report was one day (range, 0–15 days) and 616 patients (93 per cent) were seen in a hospital clinic, with 44 patients (7 per cent) reviewed in a community clinic. Clinical outcomes are summarised in Table 2.

**Table 2.** Clinical outcomes from the low-risk telescopic clinic

Clinical outcome	Frequency (*n*, (%))
**Discharge**	553 (83.8)
**Malignant head and neck cancer**	6 (0.9)
**Benign head and neck cancer (follow up booked)**	89 (13.5)
**Benign rhinology**	1 (0.2)
**Non-head and neck pathology requiring onward referral**	11 (1.7)

### The 28-day Faster Diagnosis Standard

The mean time to patients being informed whether they did or did not have cancer (Faster Diagnosis Standard) was 28.6 days (standard deviation 20.2). There was no statistically significant difference in the Faster Diagnosis Standard between patients diverted for a scan prior to review and those seen directly in the low-risk clinic (27.3 *vs* 28.9 days, *p* = 0.63). The mean time to diagnosis was shorter in patients seen in a community clinic compared with patients seen in a hospital clinic (22.6 *vs* 29.1 days, *p* < 0.01).

### Investigations

Overall, 243 patients (36.8 per cent) required some form of further investigation, with 213 patients (32 per cent) requiring imaging and 57 patients (8.6 per cent) requiring a diagnostic procedure in theatre. The frequency of investigations is summarised in Table 3.

**Table 3.** Summary of further investigations from the low-risk telescopic clinic

Investigation	Frequency (%)
**Computed tomography scan**	69 (10.5)
**Magnetic resonance imaging scan**	36 (5.5)
**Ultrasound scan**	129 (19.5)
**Theatre**	57 (8.6)

### Re-referral

Overall, 21 patients discharged from the low-risk pathway had been re-referred at the point of data collection (3.2 per cent). No head and neck cancers were diagnosed. One patient was diagnosed with an oesophageal cancer. On review of this patient's records, the flexible nasendoscopy performed during the initial referral demonstrated nothing of concern and a subsequent oesophagogastroduodenoscopy did not detect a malignancy. There were five months between the initial and subsequent two-week-wait referral (the second referral was by a local ENT consultant after seeing the patient in the private sector). The tumour was picked up on a magnetic resonance imaging scan that was arranged for persistent throat symptoms.

A second patient went on to receive a diagnosis of motor neurone disease from the neurology team after a re-referral with progressive dysphagia. Five patients received a benign head and neck diagnosis and were managed on the standard routine pathway. One patient was referred with an entirely new head and neck symptom (parotid lump rather than hoarse voice). Twelve patients were discharged at the first appointment following re-referral. Two patients were still awaiting an out-patient appointment at the time of data collection.

## Discussion

The telescopic referral pathway in place at our unit is the first of its kind in the UK to remotely triage, assess and manage two-week-wait suspected head and neck cancer referrals.^3^ Our early data demonstrated that this pathway is safe for patients and may lead to numerous benefits for the service, most notably in terms of providing flexible clinic capacity and optimising consultant time. We are now able to present data on 660 patients who have passed through the low-risk arm of this pathway, with a mean follow up of 14 months.

Head and neck cancer is the eighth most common cancer site in the UK and the number of new cases on average each year is projected to rise from around 14,000 in 2023–2025 to around 16,300 in 2038–2040.^5^ It is well established that the sensitivity of a suspected cancer pathway must be high to ensure cancers are not missed; indeed, the cancer conversion rate for head and neck cancers in the UK is in the region of 5 per cent.^6^ However, with increasing referral numbers, the 95 per cent of patients referred who do not have cancer place a significant burden on pathway capacity. As such, the concept of triaging has emerged to facilitate the identification and prioritisation of high-risk patients. Further impetus for this concept was provided by the Covid-19 pandemic.

Hardman *et al*.^7^ have presented data for over 4500 patients who underwent telephone triaging using the widely validated risk calculator adopted by our pathway, demonstrating a low risk of harm. Our pathway aims to further streamline the two-week-wait process by managing all low-risk referrals via a nurse-led clinic, with consultant review of remotely acquired images.

Our intial pilot data suggested that the described pathway was safe, but the above data collected over a two-year period gives a more robust insight into both the safety of the pathway and expected clinical outcomes. The overall cancer conversion rate in our low-risk patients of 0.9 per cent is consistent with the conversion rate observed in our pilot data (<1 per cent)^3^ and data from Hardman *et al*., who showed that the negative predictive value of a low-risk outcome from the calculator was 99.1 per cent.^7^

Whilst cancer incidence was low, incorporating remote examination by a consultant head and neck surgeon allowed us to detect six head and neck cancers that may have been missed had care been deferred based on the triage outcome alone. Two of these patients were notably young compared with the typical demographic, at 30 and 36 years.

Potential limitations in the triage process were highlighted by the patient diagnosed with a T4N0M0 supraglottic cancer, who was triaged as low risk based on the written referral from the general practitioner, but when re-scored face-to-face by the nurse practitioner was found to be high risk. Despite this, remote examination allowed a prompt diagnosis and the patient remains in remission following chemoradiotherapy.

As an exclusively low-risk cohort, our patient demographics inversely mirror what would traditionally be expected in patients with a head and neck cancer diagnosis, namely a preponderance of females compared with males (82 *vs* 18 per cent) and a relatively young mean age of 58 years. The need for subsequent investigation in just over a third of patients is in keeping with what would be expected on a standard two-week-wait pathway for head and neck cancer.^8^ Senstivity is a key parameter for such a pathway and the current data suggest there have been no missed head and neck cancers.

It should be acknowledged that only four months have passed between the most recent patients passing through the pathway and data analysis, meaning further re-referrals could emerge over the coming months. Data collection is ongoing to ensure these would be detected.

Overall, the re-referral rate was low (3.2 per cent) and looking back at the two serious diagnoses made at re-referral (oesophageal cancer and motor neurone disease), it was difficult to see how an earlier diagnosis could have been made on a traditional face-to-face pathway. In oesophageal cancer case, five months passed between the initial referral and re-referral, and an oesophagogastroduodenoscopy in this time did not make a diagnosis. The nasendoscopic images from the initial assessment were also reviewed in light of the diagnosis, but no abnormality was seen. This perhaps illustrates another potential advantage of this pathway from a documentation perspective, that all captured images are stored on the patient records system.

The Faster Diagnosis Standard was introduced in April 2020 to ensure that all patients who are referred for the investigation of suspected cancer find out within 28 days if they do or do not have a cancer diagnosis. Moving forward, this is one of the key standards against which a cancer pathway is assessed. Overall, our mean time to a patient being informed whether they did or did not have cancer fell close to this (28.6 days), but there was significant variation (range, 2–276 days).

The pathway utilises text messages as the default communication method to patients. The instant nature of text messages coupled with the short mean time between nurse practitioner review and consultant report (one day) assists with adherence to the Faster Diagnosis Standard and means, for those discharged following initial review, the limiting step was clinic capacity, where patients waited three weeks on average for review.

For patients requiring subsequent investigation, adherence to the Faster Diagnosis Standard becomes increasingly challenging, but there are numerous factors at play. Indeed, for patients with the longest time to Faster Diagnosis Standard, non-attendance at appointments was a key issue. Clearly this is an area that needs to be optimised moving forward; options may include expansion of the low-risk pathway to increase clinic capacity and provide a more timely initial review.

- The two-week-wait suspected head and neck cancer pathway is under considerable strain from a combination of increasing referrals, pandemic recovery and the new 28-day Faster Diagnosis Standard
- A novel telescopic pathway has been implemented at our unit that utilises high-quality mobile imaging alongside secure store-and-forward technology to streamline the two-week-wait head and neck cancer pathway
- This study reports on 660 patients who have passed through the new pathway with a mean follow up of 15 months
- Results suggest that low-risk head and neck cancer referrals can be safely managed in a nurse-led clinic for recorded examination with asynchronous consultant-led management
- Further work is required to investigate other uses of a nurse-led clinic, such as follow-up examination, and optimisation is required to ensure adherence to the 28-day Faster Diagnosis Standard

Our data demonstrate that a combination of risk stratification and asynchronous telescopic assessment facilitates management of suspected head and neck cancer referrals and many of the concepts incorporated is likely to become more prevalent in the specialty moving forward. The EVEREST-HN research programme is already underway and looks to utilise ‘big data’ to refine the triage process further to identify high-risk patients for targeted investigation prior to their hospital appointment.^8^ It will also serve to reassure the worried well sooner.

Interesting work is emerging on the use of artificial intelligence (AI) to augment the triage process whereby patients are triaged via automated voice recognition,^9^ which may have benefits in terms of staff utilisation and reducing human errors. However, no data have yet been published. AI may also have potential for the detection of early-stage laryngeal cancer based on endoscopically acquired images.^10^ The use of other diagnostic adjuncts, such as narrow band imaging, may also enhance a remote assessment pathway as their application becomes more widespread.

While our pathway includes a trained nurse practitioner to examine patients, the use of senior speech and language therapists as part of a similar pathway has also been reported. Butler *et al*.^11^ have published data from a speech and language therapist-led two-week-wait clinic that has the advantage of employing speech and language therapist expertise to provide a therapeutic service to patients with voice symptoms in addition to the primary aim of cancer detection. Whilst the speech and language therapist team operate with some autonomy, the care of all patients in this study was overseen by a consultant head and neck surgeon, as is the case with our model.

## Conclusion

Low-risk head and neck cancer referrals can be safely managed in a nurse-led clinic for recorded examination with asynchronous consultant-led management. Whilst a small number of patients are re-referred, to date there have been no missed head and neck cancers. Further work is required to investigate other uses of a nurse-led clinic, such as follow-up examination, and optimisation is required to ensure adherence to the 28-day Faster Diagnosis Standard.
